# The Neural Correlates of Visual and Auditory Cross-Modal Selective Attention in Aging

**DOI:** 10.3389/fnagi.2020.498978

**Published:** 2020-11-12

**Authors:** Franziska Rienäcker, Pascal W. M. Van Gerven, Heidi I. L. Jacobs, Judith Eck, Caroline M. Van Heugten, Maria J. S. Guerreiro

**Affiliations:** ^1^Department of Neuropsychology and Psychopharmacology, Faculty of Psychology and Neuroscience, Maastricht University, Maastricht, Netherlands; ^2^Department of Educational Development and Research, Faculty of Health, Medicine and Life Sciences, School of Health Professions Education (SHE), Maastricht University, Maastricht, Netherlands; ^3^Department of Psychiatry and Neuropsychology, Faculty of Health, Medicine and Life Sciences, School of Mental Health and Neuroscience, Maastricht University, Maastricht, Netherlands; ^4^Division of Nuclear Medicine and Molecular Imaging, Department of Radiology, Massachusetts General Hospital/Harvard Medical School, Boston, MA, United States; ^5^Department of Cognitive Neuroscience, Faculty of Psychology and Neuroscience, Maastricht University, Maastricht, Netherlands; ^6^Biological Psychology and Neuropsychology, Institute for Psychology, University of Hamburg, Hamburg, Germany

**Keywords:** selective attention, sensory modality, aging, whole-brain fMRI, top-down modulation

## Abstract

Age-related deficits in selective attention have been demonstrated to depend on the sensory modality through which targets and distractors are presented. Some of these investigations suggest a specific impairment of cross-modal auditory selective attention. For the first time, this study is taking on a whole brain approach while including a passive perception baseline, to investigate the neural underpinnings of selective attention across age groups, and taking the sensory modality of relevant and irrelevant (i.e., distracting) stimuli into account. Sixteen younger (mean age = 23.3 years) and 14 older (mean age = 65.3 years), healthy participants performed a series of delayed match-to-sample tasks, in which participants had to selectively attend to visual stimuli, selectively attend to auditory stimuli, or passively view and hear both types of stimuli, while undergoing 3T fMRI. The imaging analyses showed that areas recruited by cross-modal visual and auditory selective attention in both age groups included parts of the dorsal attention and frontoparietal control networks (i.e., intraparietal sulcus, insula, fusiform gyrus, anterior cingulate, and inferior frontal cortex). Most importantly, activation throughout the brain did not differ across age groups, suggesting intact brain function during cross-modal selective attention in older adults. Moreover, stronger brain activation during cross-modal visual vs. cross-modal auditory selective attention was found in both age groups, which is consistent with earlier accounts of visual dominance. In conclusion, these results do not support the hypothesized age-related deficit of cross-modal auditory selective attention. Instead, they suggest that the underlying neural correlates of cross-modal selective attention are similar in younger and older adults.

## Introduction

Selective attention enables a person to attend to relevant stimuli in the environment while ignoring irrelevant stimuli. The strong need for this ability becomes apparent by considering the limited human processing capacity (Miller, 1956). Because an individual can only attend to a limited portion of the environment, incoming stimuli need to be filtered. In contrast to bottom-up attentional modulation, which happens when an individual's attentional focus is passively drawn toward environmental, incoming stimuli, selective attention requires active selection, that is, facilitation of relevant and suppression of irrelevant stimuli, which is referred to as “top-down” attentional modulation. This modulation takes place at two levels: (1) relevant stimuli are enhanced over other stimuli to facilitate processing of those stimuli and (2) processing of irrelevant stimuli is inhibited to avoid distraction. Research into healthy cognitive aging has primarily focused on the latter mechanism: inhibition. A variety of common cognitive challenges in old age, such as memory decline, are thought to be the result of deficits in the inhibition of task-irrelevant information. This so-called inhibitory deficit hypothesis was originally proposed by Hasher and Zacks (1988). Since then, many studies have offered support for the notion that older individuals are less effective at inhibiting irrelevant information than their younger counterparts (e.g., Zacks and Hasher, 1994; Lustig et al., 2001).

Notably, the majority of studies investigating selective attention in aging employed visual tasks with visual distraction, whereas only a small number of studies involved auditory targets and distractors. Only a few studies employed cross-modal selective attention paradigms (for a review, see: Guerreiro et al., 2010; Van Gerven and Guerreiro, 2016), for instance visual or auditory tasks with distraction from the other modality. This is remarkable, as in most everyday situations relevant and irrelevant stimuli are conveyed through multiple modalities. At least three studies investigated performance of younger and older participants on a working memory task that involved all possible combinations of visual and auditory attention and distraction (Guerreiro and Van Gerven, 2011; Guerreiro et al., 2013; Rienäcker et al., 2018). In all of these studies, older adults were specifically impaired in conditions of auditory selective attention with visual distraction. This suggests that the inhibitory deficit hypothesis applies to cross-modal auditory selective attention.

The neural correlates of this specific age-related deficit have however remained unclear. In the unimodal visual domain, Gazzaley and colleagues have shown age-related impairments in the neural suppression of irrelevant stimuli in visual category-selective cortical areas. On the other hand, enhancement of relevant information has been found to be intact in healthy older, as compared to younger participants (Gazzaley et al., 2005b). Looking beyond sensory brain areas, Geerligs et al. (2014) demonstrated an involvement of the dorsal attention network (DAN) and the frontoparietal control network (FPCN)—including the dorsolateral prefrontal cortex (DLPFC), parts of the parietal cortex, the rostrolateral prefrontal cortex (RLPFC), and the cerebellum—in age-related selective attention. Compared to younger participants, older participants demonstrated increased activation of these areas during unimodal visual attention—as well as increased connectivity to somatosensory regions—during the detection of relevant target stimuli.

In the domain of cross-modal selective attention, functional magnetic resonance imaging (fMRI) and electroencephalogram (EEG) studies comparing top-down modulation across age groups, have revealed brain responses that appear to be unaffected by normal aging (e.g., Mishra and Gazzaley, 2013; Guerreiro et al., 2014). Mishra and Gazzaley (2013) demonstrated age-equivalent early event-related potentials (ERPs)—such as the P1 and N1—in visual and auditory cortices during cross-modal selective attention. Guerreiro et al. (2015) investigated top-down modulation during both uni- and cross-modal visual and auditory distraction in category-specific brain areas in a sample of healthy younger and older adults. The employed memory task, which involved relevant and irrelevant, visual, and auditory information (adapted from Gazzaley et al., 2005a), showed age-independent visual enhancement, as well as, and most importantly, age-independent suppression of visual cortical processing during auditory selective attention, relative to a perceptual baseline condition. These results are consistent with earlier accounts of age-equivalent enhancement and suppression of auditory and visual information in cross-modal paradigms (Mishra and Gazzaley, 2013; Guerreiro et al., 2014), but stand in stark contrast with earlier uni-modal selective attention studies that found clear age-differences in visual suppression (e.g., Gazzaley et al., 2005b).

Further evidence for age effects in cross-modal selective attention come from a small proportion of neuroimaging studies that have also looked beyond sensory-specific cortical areas. These studies indicate a different pattern, depending on modality. For example, Townsend et al. (2006) studied cortical modulation during cross-modal auditory and visual distraction in younger and older adults. Using a whole-brain analysis, they demonstrated similar brain areas to be involved in cross-modal auditory attention across age groups, which is in line with the aforementioned region of interest (ROI) results on cross-modal selective attention (e.g., Guerreiro et al., 2014). In older adults, however, more widespread areas of the inferior frontal gyrus, the left insula, and left fusiform gyrus were recruited during cross-modal visual attention. Different patterns across modalities have also been demonstrated in early ERP components. The P1 and N1 components are commonly modulated by attentional processes. Top-down neural modulation, reflected by these components, was found to be unaffected by aging in auditory distraction conditions (Čeponienė et al., 2008). Moreover, in older participants, P1 and N1 components were diminished in visual distraction conditions, as compared to younger participants. In sum, the results of these cross-modal paradigms looking beyond sensory and category specific cortical regions point toward an important role of sensory modality in age-related differences in selective attention.

In contrast to the aforementioned ROI studies, most whole-brain investigations did not use a passive perceptual baseline, that is, they did not base their estimations of neural modulation on a comparison between selective attention conditions and a passive viewing or listening baseline, but directly compared brain activation across groups or conditions. This might be less favorable, as it does not allow for any interpretation regarding the direction of activation differences (i.e., enhancement or suppression relative to baseline).

In sum, there is little research into the neural underpinnings of cross-modal selective attention deficits in aging. Research that is available is methodologically diverse and results are contradictory. Neuroimaging investigations focusing on sensory-specific brain responses have demonstrated age-equivalent top-down modulation during cross-modal visual and auditory selective attention (Mishra and Gazzaley, 2013; Guerreiro et al., 2014, 2015). This may imply that the earlier observed sensory-specific performance deficits (e.g., Guerreiro et al., 2013) are the result of age-related differences in the modulation of other, higher-order, cortical regions. Studies undertaking a whole-brain approach indeed point toward possible age differences in cross-modal attentional modulation, depending on sensory modality, however, without employing a perceptual baseline. Therefore, the aim of the current study was to investigate cortical activity beyond sensory areas, in response to cross-modal visual and auditory selective attention, relative to a perceptual baseline, a combination of conditions that has never been investigated before. We aimed to examine whether this activity differs between age groups, and if these differences are modulated by sensory modality. Based on previous studies investigating cross-modal selective attention (Townsend et al., 2006; Čeponienė et al., 2008; Mishra and Gazzaley, 2013), we hypothesized that, similar to unimodal visual selective attention, frontoparietal areas are involved in cross-modal selective attention, that activity in these areas is more widespread in older individuals, and that this age-effect might depend on the sensory modality of the distraction.

## General Methods

### Participants

Data of 16 younger (aged 20–29 years, *M* = 23.3, *SD* = 3.0, 9 women) and 14 older participants (aged 60–71 years, *M* = 65.3, *SD* = 4.1., 10 women) was employed for this study (Guerreiro et al., 2015). There was no significant association between age group and sex, χ^2^(1) = 0.74, *p* = .389, indicating that sex distribution did not differ between younger and older adults. All participants were right-handed, free of major physical or psychiatric illnesses, and reported having normal or corrected-to-normal vision and hearing. Older participants were cognitive healthy, as indicated by a score of 18 or higher on the Cognitive Screening Test (De Graaf and Deelman, 1991). In total, 17 younger and 21 older participants went through the testing procedure. Data of one younger and seven older participants were excluded because of excessive head movement (> 3.5 mm) during scanning, resulting in a final sample of 16 younger and 14 older participants. Recruitment was done through advertisements on local bulletin boards. This study was approved by the Ethics Review Committee Psychology and Neuroscience. Written informed consent was obtained from all participants before testing.

### Task and Procedure

**Figure 1:**
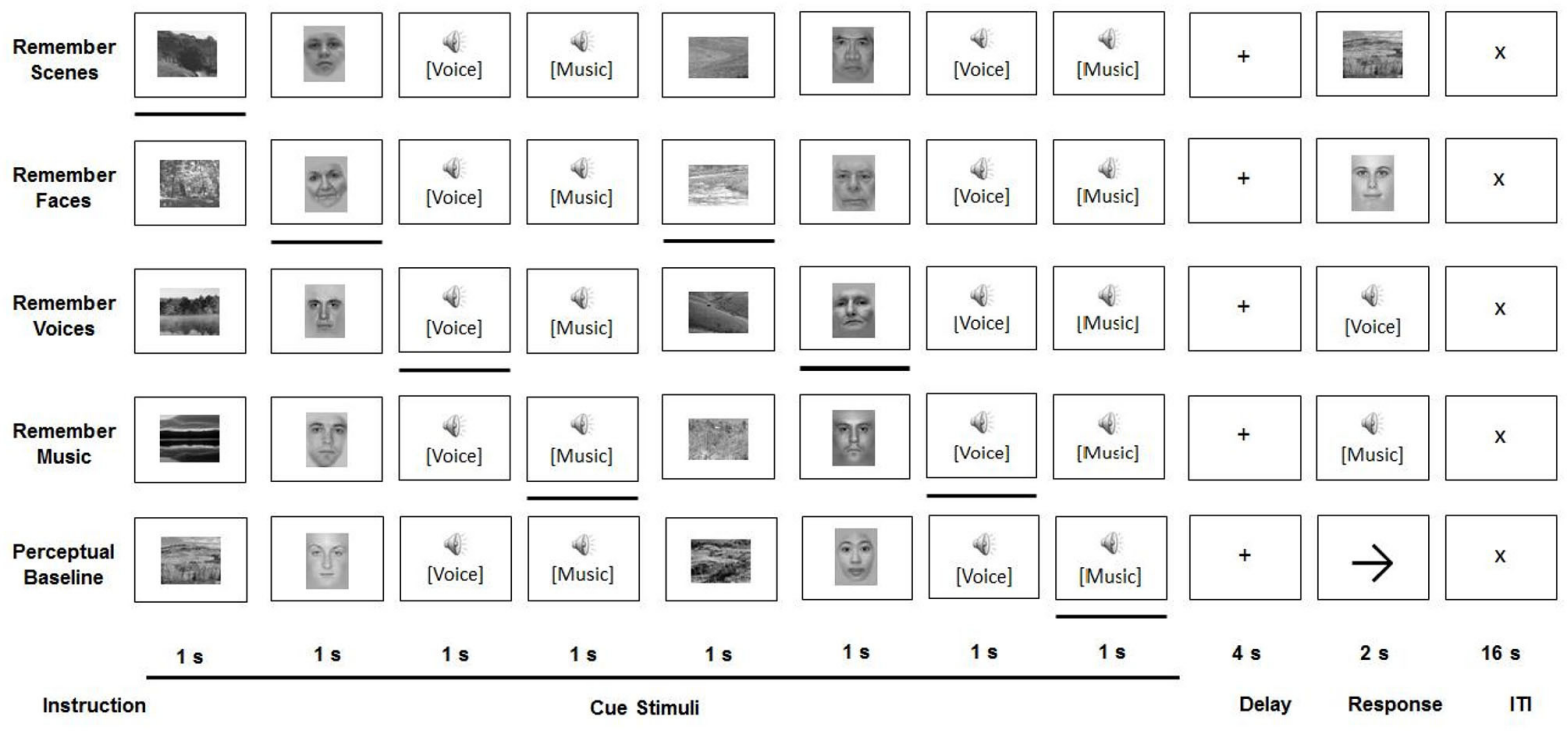
Experimental paradigm.

Younger and older participants were asked to perform a working memory-based selective attention task, developed by Gazzaley et al. (2005a) and adapted by Guerreiro et al. (2015), while undergoing fMRI (see Figure 1).

All task conditions involved a sequential presentation of stimuli from four categories: faces, scenes, voices, and music. Face stimuli (neutral portrait pictures of male and female adults) and scene stimuli were presented in black-and-white, at a resolution of 225 × 300 pixels, in the center of a computer screen. The screen was projected via a mirror to be visible from inside the MRI scanner. Voice stimuli included Portuguese trisyllabic words, recorded by male and female native speakers. In order to prevent semantic processing of the words, only participants without understanding of the Portuguese language were included. Music stimuli were recorded at a sampling rate of 44 kHz and presented with normalized amplitude with a duration of 800 ms.

All participants performed five tasks—two visual attention tasks, two auditory attention tasks, and a perceptual baseline task—in a counterbalanced order. The setup of the trials was identical for each of these conditions. During a cue period, two stimuli of each of four categories (faces, scenes, voices, and music) were sequentially presented in a pseudo-random order, whereby each was shown for 800 ms, followed by an inter-stimulus interval of 200 ms. After presentation of these eight stimuli, and following a 4000 ms delay period, a probe stimulus from the relevant category was displayed, to which a response had to be given during a 2000 ms response interval. Each condition—which was presented in separate experimental runs—consisted of 24 such trials. The conditions only differed in the instructions given. During the four selective attention conditions (remember faces, remember scenes, remember voices, and remember music), one stimulus category presented in the cue period had to be attended to, while the other three had to be ignored. During the presentation of the probe stimulus from this category, the participant was asked to indicate whether or not this stimulus had been shown in the cue period. Participants were instructed to respond as accurately as possible with a button press (yes/no). In the perceptual baseline condition, participants were asked to passively view and listen to the stimuli, without attempting to memorize any of them. Subsequently, an arrow pointing left or right was displayed instead of a probe stimulus. Participants were instructed to give a left/right response, based on the direction of this arrow.

### Analysis of Behavioral Data

Behavioral data were analyzed using IBM SPSS Statistics version 22. Accuracy data (in %) of all conditions was submitted to a 2 (Age Group: younger, older) × 5 (Condition: passive baseline, remember scenes, remember faces, remember voices, remember music) repeated-measures analysis of variance (ANOVA). Age Group was the between- and Condition the within-groups factor. Results were considered significant at *p* < 0.05. A Greenhouse-Geisser correction was applied if the assumption of sphericity was violated. To correct for multiple comparisons, a Bonferroni correction was applied.

### MRI Acquisition

All participants underwent 3-T (f)MRI scanning in a Siemens Allegra head scanner (Siemens Allegra, Erlangen, Germany) at the Maastricht Brain Imaging Center (M-BIC), employing a quadrature birdcage coil.

T1-weighted anatomical images were recorded with an ADNI MPRAGE sequence, covering the whole brain (192 sagittal slices; matrix size = 256 × 256; voxel dimensions = 1 × 1 × 1 mm; repetition time [TR] = 2250 ms; echo time [TE] = 2.6 ms; flip angle [FA] = 9°). Anatomical images were recorded after two of the five selective attention task conditions, to allow participants to rest in between. T2^*^ 2D-functional images were acquired with an EPI sequence (TR = 2000 ms; TE = 30 ms; FA = 90°; slice thickness = 3.5 mm; 32 axial slices; matrix size = 64 × 64; field of view = 224 × 224).

A significant concern in fMRI studies, especially those involving the presentation of auditory stimuli, is that the acoustic noise generated by standard EPI sequences may render participants unable to adequately perceive the auditory stimuli, rendering the interpretation of neural responses problematic (e.g., Peelle, 2014). Although the use of sparse imaging has been proposed to bypass this problem, we used a standard EPI sequence for two interrelated reasons: The present paradigm constituted a replication and extension of the paradigm originally developed by Gazzaley et al. (2005a), so not only did we want to keep it as close to the original as possible, but also modifying it for use with a sparse sequence would have led to a prohibitively long task duration, considering our five experimental conditions. To ensure, however, that participants were able to adequately perceive the auditory stimuli, we adjusted the intensity of the auditory stimuli prior to task performance to equalize the perceived loudness across participants. This was done by presenting a sample of voice and music stimuli while participants were in the scanner with earplugs and headphones and the EPI sequence was running, in order to replicate the background noise throughout the tasks. The intensity of the auditory stimuli was increased or decreased until a hearing level was reached that was both audible and comfortable for each participant.

### fMRI Data Analysis

In contrast to the previously published work (Guerreiro et al., 2015), this investigation takes on a whole brain approach instead of a specific ROI approach. This enables the detection of activation (differences) in cross-modal selective attentional modulation in brain areas beyond the previously investigated sensory and category selective regions of interest. This is an important addition, as attention processes are known to involve especially prefrontal and parietal areas.

All acquired (f)MRI data were analyzed with BrainVoyager, version 20.6.2.3266 (BVQX 3.6.2) (Brain Innovation, Maastricht, the Netherlands). Pre-processing of functional data included head motion correction, temporal high-pass filtering, spatial smoothing (6 mm), and slice scan time correction using sinc interpolation. The first two functional volumes were discarded. Functional data were co-registered with the intra-session structural data set and transformed into Montreal Neurological Institute (MNI) space.

Functional data were analyzed with a single-subject general linear model (GLM) with separate predictors for the cue, delay, and response period for each of the experimental conditions and runs (e.g., passive baseline: cue, remember faces: delay, etc.). All regressors were convolved with a double gamma hemodynamic response function before entering them in the model. Confounding predictors included movement parameters of all directions, as well as predictors for trials with incorrect responses. For the subsequent group analyses, multi-subject random effects GLMs were computed with predictors of the cue period at a whole-brain level, involving contrasts for: cross-modal visual attention vs. passive baseline [remember faces (RF) + remember scenes (RS) > passive baseline (PB)], cross-modal auditory attention vs. passive baseline (remember music (RM) + remember voices (RV) > PB), and cross-modal visual attention vs. cross-modal auditory attention (RF + RS > RM + RV). All active conditions include cross-modal, as well as uni-modal attention and distraction. Taking together both visual, and both auditory categories (i.e., RF + RS and RM + RV), we expect that uni-modal enhancement and suppression is canceled out, to remain with an index of cross-modal attention only. All specified contrasts were balanced and compared between and across age groups. The obtained statistical maps were Bonferroni-corrected at *p* < 0.05 (two-sided). Peak activations were extracted from each cluster. The MNI coordinates of the cluster peaks were associated with anatomical and functional brain regions with guidance of the Yale BioImage Tal/MNI to Brodmann tool (based on Lacadie et al., 2008).

## Results

### Behavioral Results

**Figure 2:**
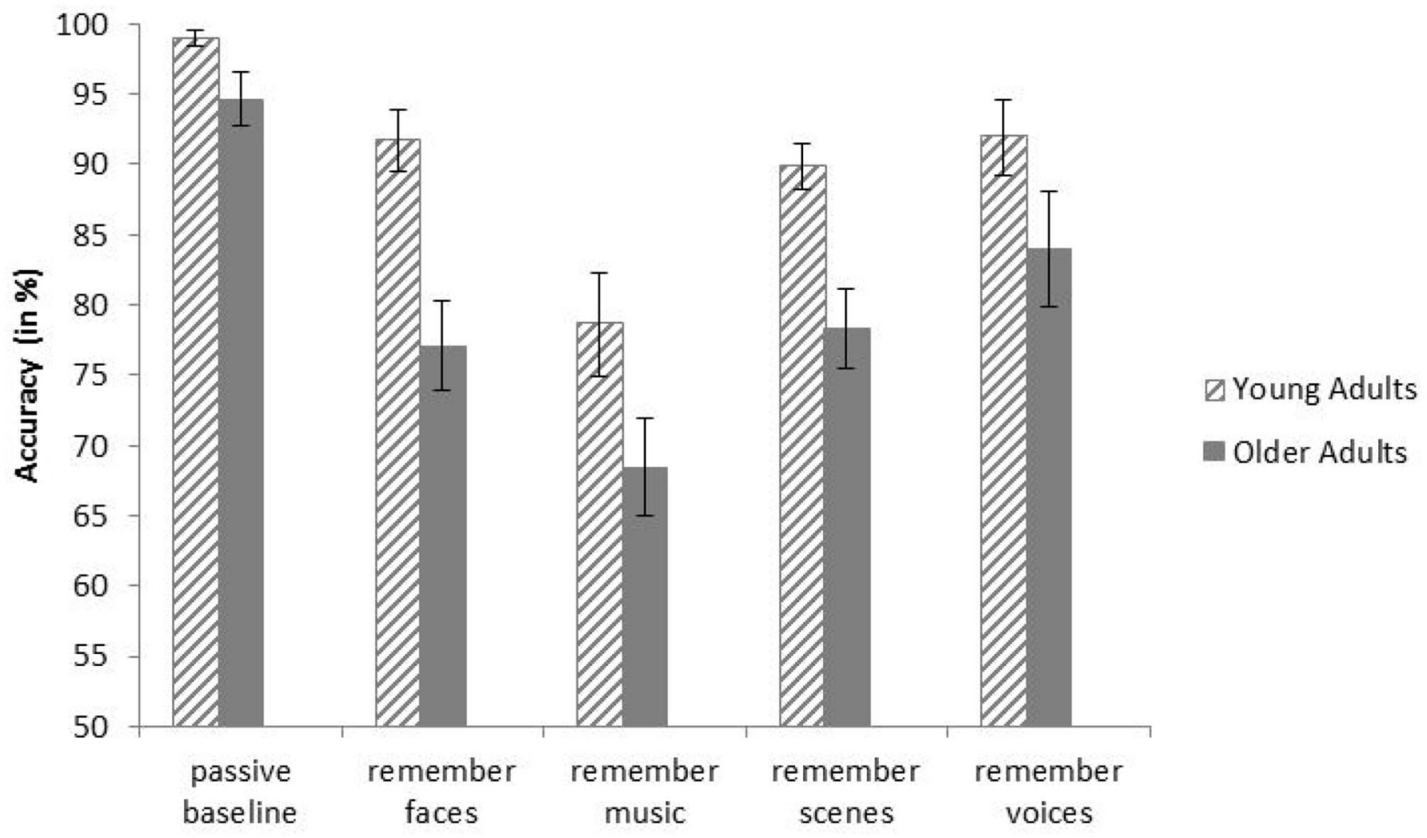
Mean accuracies of younger and older adults in all conditions of the behavioral task. Error bars indicate one standard error of the mean.

There was a significant main effect of Age Group on task accuracy (% correct responses), *F*_(1, 28)_ = 18.18, *p* < 0.001, η_p_^2^ = 0.39, indicating that the younger participants were significantly more accurate than the older participants (*M* = 90.20%, *SD* = 5.15, and *M* = 80.48%, *SD* = 7.29, respectively). Furthermore, there was a main effect of Condition, *F*_(3.01, 84.40)_ = 21.80, *p* < 0.001, η_p_^2^ = 0.44. Pairwise comparisons revealed that, across age groups, participants were significantly more accurate in the baseline condition than in all selective attention conditions (*p*s < 0.001). There was no significant interaction between Age Group and Condition, *F*_(3.01, 84.40)_ = 1.16, *p* = .330, η_p_^2^ = 0.04, indicating that the condition effect was independent of Age Group. Means and standard errors of both age groups and all conditions are displayed in Figure 2.

### fMRI Results

#### Cross-Modal Visual Selective Attention

**Figure 3:**
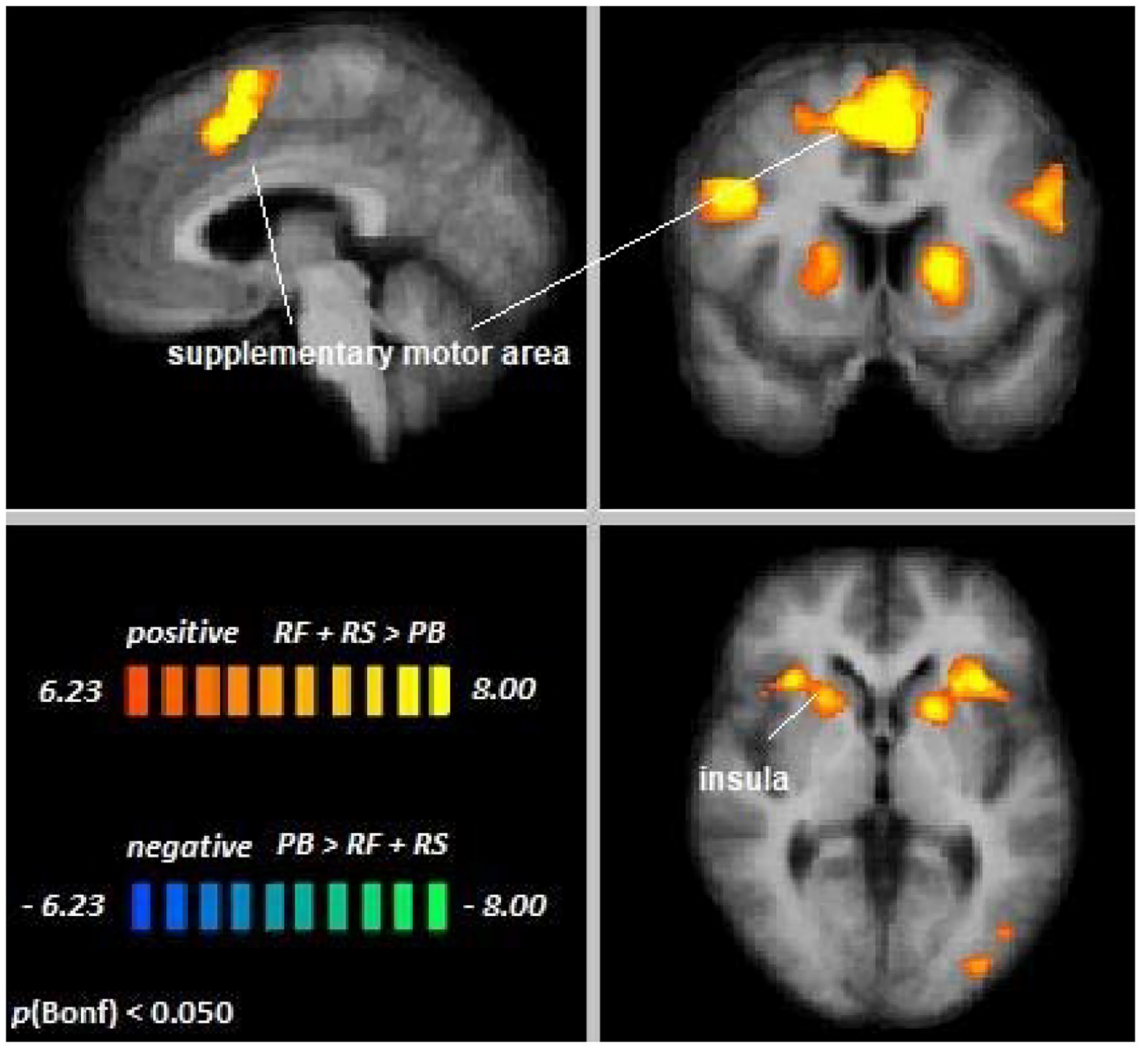
Remember faces and remember scenes vs. passive baseline. An overview of significant clusters for the contrast RF + RS > PB. Sagittal, coronal, and axial planes are shown at *x* = 3, *y* = 6, *z* = 2. Active clusters indicate voxels with increased activation in visual selective attention conditions relative to baseline.

Across age groups, higher activation during visual attention conditions relative to the perceptual baseline was observed in the left supplementary motor area, the premotor cortex, lateral parietal cortex, parts of the inferior frontal gyrus, right insula, left fusiform gyrus, and the left occipital visual association cortex (VAC) (see Figure 3). For all cluster peaks, associated Brodmann areas (BAs), and MNI coordinates, see Table 1.

**Table 1:** Cluster location and peaks across age groups (RF + RS > PB).

			**MNI coordinates**				
**Location**	**Hemisphere**	**BA**	***x***	***y***	***z***	**Peak *t***	**Size**
Supplementary motor area	LH (medial)	6	0	11	49	12.39	26511
Intraparietal sulcus (IPS)	LH	7	−21	−67	52	12.34	20930
	RH	7	21	−61	58	11.25	9095
Inferior frontal gyrus	LH	45	−33	20	7	12.24	6237
Premotor cortex	LH (lateral)	6	−45	−1	37	11.75	5561
Insula	RH	13	33	23	4	9.90	3284
Angular gyrus	RH	39	33	−76	22	8.78	11330
Fusiform gyrus	LH	37	−51	−64	−8	8.54	3927
Occipital VAC	LH	19	−36	−85	−2	7.47	720

*LH, left hemisphere; RH, right hemisphere; BA, Brodmann area; RF, remember faces; RS, remember scenes; PB, passive baseline; VAC, visual association cortex. ps < 0.0001, Bonferroni correction was applied. Clusters are displayed, sorted by t value, cluster sizes in number of 1 mm^3^ voxels*.

The contrast Young Adults [RF + RS > PB] > Older Adults [RF + RS > PB] revealed no differences between age groups, suggesting that top-down modulation in the setting of cross-modal visual selective attention was the same for younger and older adults. Single-subject data corroborated this notion in the regions showing significant attentional modulation during cross-modal visual attention (see Supplementary Figure 1).

#### Cross-Modal Auditory Selective Attention

**Figure 4:**
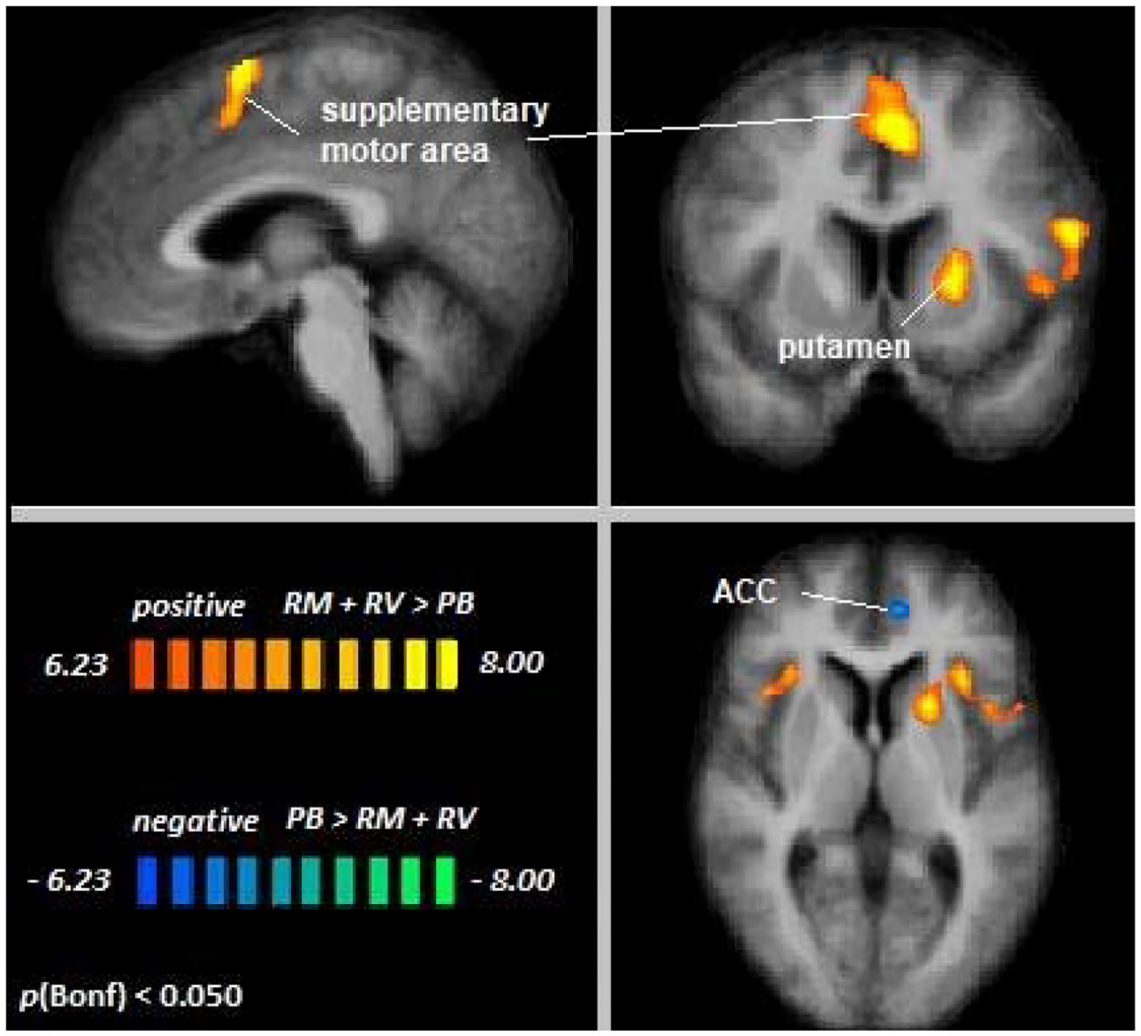
Remember music and remember voices vs. passive baseline. An overview of significant clusters for the contrast RF + RS > PB. Sagittal, coronal, and axial planes are shown at *x* = 3, *y* = 6, *z* = 2. Orange to yellow clusters indicate voxels with greater activation in auditory selective attention conditions relative to baseline. Blue to green clusters reveal voxels with lower activation in auditory selective attention conditions relative to baseline.

Across age groups, higher activation during auditory attention conditions relative to the perceptual baseline was observed in the right supplementary motor area, left inferior frontal gyrus, left putamen, and right insula, whereas lower activation was observed in the anterior cingulate cortex (see Figure 4). For all cluster peaks, associated BAs, and MNI coordinates, see Table 2.

**Table 2:** Cluster location and peaks across age groups (RM + RV > PB).

			**MNI coordinates**				
**Location**	**Hemisphere**	**BA**	***x***	***y***	***z***	**Peak *t***	**Size**
Supplementary motor area	RH (medial)	6	3	8	61	10.42	6160
Inferior frontal gyrus	LH	44	−57	11	16	9.32	2027
Putamen	LH	49	−21	11	4	8.54	1432
Insula	RH	13	33	23	4	7.34	393
Anterior cingulate cortex	LH	32	−9	47	4	−7.30	401

*LH, left hemisphere; RH, right hemisphere; BA, Brodmann area; RV, remember voices; RM, remember music; PB, passive baseline. ps < 0.0001, Bonferroni correction was applied. Clusters are displayed, sorted by t value, cluster sizes in number of 1 mm^3^ voxels*.

The contrast Young Adults [RM + RV > PB] > Older Adults [RM + RV > PB] revealed no differences between age groups, suggesting that top-down modulation in the setting of cross-modal auditory selective attention was the same for younger and older adults. Single-subject data confirmed this notion in the regions showing significant attentional modulation during cross-visual auditory attention (see Supplementary Figure 2).

#### Differences in Cross-Modal Selective Attention Across Modalities

**Figure 5:**
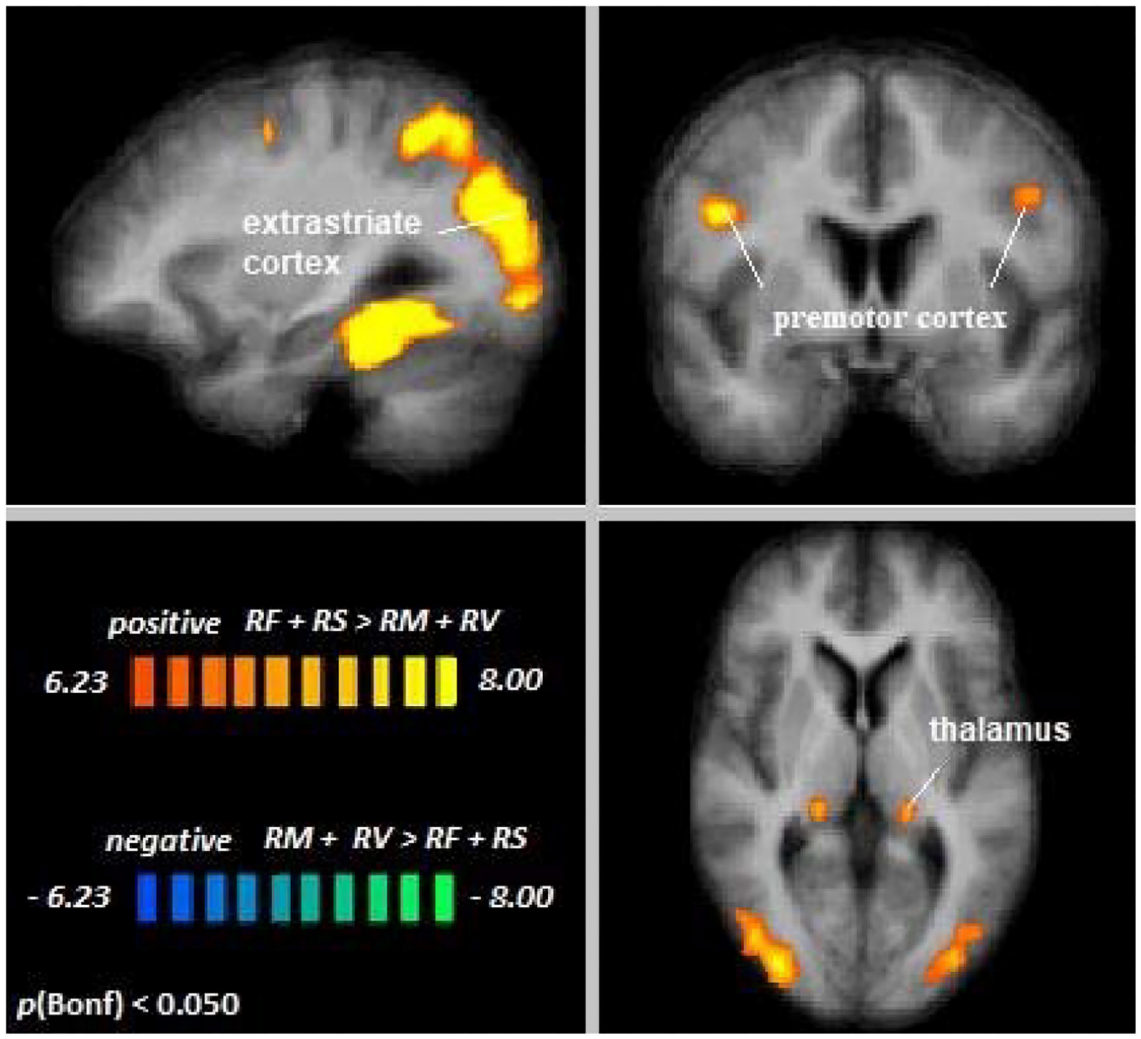
Remember faces and remember scenes vs. remember music and remember voices. An overview of significant clusters for the contrast RF + RS > RM + RV. Sagittal, coronal, and axial planes are shown at *x* = 3, *y* = 6, *z* = 2. Active clusters show voxels with increased activation in visual relative to auditory selective attention conditions.

Across age groups, higher activation during cross-modal visual selective attention relative to cross-modal auditory selective attention was observed in the right fusiform gyrus, the extrastriate cortex, premotor cortex, posterior cingulate cortex, and left thalamus (see Figure 5). For all cluster peaks, associated BAs, and MNI coordinates, see Table 3.

**Table 3:** Cluster location and peaks across age groups (RF + RS > RM + RV).

			**MNI coordinates**				
**Location**	**Hemisphere**	**BA**	***x***	***y***	***z***	**Peak *t***	**Size**
Fusiform gyrus	RH	37	36	−40	−17	14.87	36780
Extrastriate cortex	LH	19	−36	−85	22	14.25	36433
Premotor cortex	RH	6	48	5	28	9.80	1052
	LH		−45	2	31	7.23	331
Posterior cingulate cortex	RH	23	15	−52	10	8.50	591
Thalamus	LH	n/a	−18	−31	1	8.09	498

*LH, left hemisphere; RH, right hemisphere; BA, Brodmann area; RF, remember faces; RS, remember scenes; RM, remember music; RV, remember voices. ps < 0.0001, Bonferroni correction was applied. Clusters are displayed, sorted by t value, cluster sizes in number of 1 mm^3^ voxels*.

The contrast Young Adults [RF + RS > RM + RV] > Older Adults [RF + RS > RM + RV] revealed no differences between age groups, suggesting that differences in top-down modulation across modalities were the same for younger and older adults. Single-subject data corroborated this notion in the regions showing significant differences in attentional modulation across sensory modalities (see Supplementary Figure 3).

## Discussion

The aim of the current investigation was to identify cortical areas involved in visual and auditory cross-modal selective attention and understand how activation patterns in these areas differ with age or sensory modality. Findings from previous studies provided inconsistent results, possibly due to methodological differences. By incorporating a perceptual baseline condition, we have now been able to show recruitment of several brain regions known to be involved in attentional regulation, such as the frontoparietal, and fronto-subcortical areas of the frontoparietal control network, the dorsal attention network, and the salience network. A variety of brain regions, including visual specific, as well as visual unspecific areas, such as the posterior cingulate cortex, have moreover been found to be more heavily recruited under conditions of cross-modal visual, as compared to auditory selective attention in both age groups. Recruitment of brain resources during cross-modal selective attention did not differ across age groups, suggesting age-independent top-down modulation during cross-modal selective attention.

Independent of age, large areas of the left and right supplementary motor cortex, the inferior frontal cortex, and the insula have been demonstrated to show increased activation during visual and auditory attention relative to a perceptual baseline condition. Activation in the premotor and motor areas may reflect response planning in anticipation of the button press that was required by the tasks (cf. Nachev et al., 2008). In contrast to the perceptual baseline, participants may have prepared for a motor response already during the stimulus presentation. Moreover, during conditions of visual selective attention, activation of regions commonly related to visual processing, such as the occipital visual association cortex (VAC) and the bilateral intraparietal sulcus (IPS), was found to be enhanced relative to the perceptual baseline. Especially the insula and IPS have been established as important players in various, predominantly visual, attentional processes and as nodes in the FPCN and the salience network (Corbetta and Shulman, 2002). The current results suggest that this role extends to cross-modal visual and auditory selective attention. The anterior cingulate cortex was also observed to be affected by top-down modulation during auditory selective attention, demonstrating lower activation in the auditory selective attention conditions than in the perceptual baseline condition, possibly reflecting default mode network deactivation during the active task conditions. When directly comparing cross-modal visual and auditory selective attention, our results suggest that visual processing might require more brain resources than auditory processing. That is, no cortical area has been observed to be more involved in auditory than in visual selective attention, but visual selective attention seems to more heavily rely on a variety of brain areas, as indicated by positive activation clusters resulting from the visual > auditory comparison. These areas include the bilateral premotor cortex, the posterior cingulate cortex, and regions related to visual attentional processing (i.e., the right fusiform gyrus, occipital extrastriate areas, thalamus, and posterior cingulate cortex), representing important nodes of several neural networks related to bottom-up and top-down selective attention (Leech and Sharp, 2014). In light of these results, attention networks might be more heavily used in visual than in auditory selective attention. This finding is in accordance with the visual dominance hypothesis. That is, visual processing has repeatedly been found to be dominant over auditory (and tactile) processing, on a behavioral, as well as neurophysiological level (Posner et al., 1976; Colavita and Weisberg, 1979). In line with this, recent research has found healthy young participants to enhance top-down processing of visual selective attention in several brain regions in the presence of auditory distraction, whereas processing of relevant auditory stimuli was suppressed by irrelevant visual stimuli (Yan et al., 2015). The present results substantiate this account of visual dominance in the context of cross-modal selective attention and offer a possible neural underpinning of the previously observed behavioral findings (e.g., Posner et al., 1976).

Contrary to our expectations, top-down modulation during cross-modal visual and auditory selective attention did not differ between age groups. Instead, our results suggest that top-down modulation during cross-modal visual and auditory selective attention is intact in older adults. This is consistent with earlier ROI results (Guerreiro et al., 2015), which demonstrated that older adults effectively enhance cortical processing of relevant visual information and suppress cortical processing of irrelevant visual information in the setting of auditory selective attention in a visual category-selective brain area (the parahippocampal place area). These accounts of age-equivalent top-down processing stand in contrast with reports of increased recruitment of cortical resources in aging, possibly representing a compensatory mechanism (Geerligs et al., 2014; Grady et al., 2016). Čeponienė et al. (2008), as well as Townsend et al. (2006), found age-independent brain responses during attention to one modality, but age-related differences in brain responses during attention to the other, representing a modality specific asymmetry depending on whether relevant or irrelevant information is visual or auditory. While, in line with this, the current results do support differential processing of visual and auditory cross-modal selective attention, previously reported age differences have not been replicated.

The observed lack of age differences in the present results may be explained by the following three notions. First, research focusing on unimodal visual selective attention suggests an important dissociation between enhancement and suppression in selective attention (Gazzaley et al., 2005a; Haring et al., 2013). Haring et al. (2013) demonstrated that younger and older participants did not differ in their overall modulation (as measured with EEG) between “ignore” and “attend” conditions. However, in comparison to younger participants, older individuals did not suppress activation below baseline in the “ignore” condition, but did enhance activation in the “attend” conditions. Similar patterns were observed by Gazzaley et al. (2005b). While it is still unclear whether these results also apply to cross-modal selective attention, the findings suggest a dissociation between enhancement and suppression in selective attention. The current study can only give a common index of cross-modal selective attention, including both mechanisms, especially concerning higher-order, sensory-unspecific brain areas. Therefore, it may be beneficial for future investigations to employ a paradigm that allows independent analysis of these processes.

A second factor that may be important to explore in more detail is age. Recent work indicated that sub-groups of older adults may differ in overall neural modulation, depending on whether they are at the higher or lower end of the age range (Manan et al., 2018). Brain activation during unimodal auditory selective attention was increased in adults until the age of 47, possibly reflecting a compensation mechanism, but decreased for adults beyond that age. This was interpreted as a result of advanced neural disintegration, possibly due to age-related cerebral volumetric changes. Combining “compensators” and “deteriorators” in one group may dilute these associations and could result in an overall unchanged index of neuronal modulation between younger and older participants. Future studies should therefore adopt a lifespan approach to investigate nonlinear relationships between attention and neural recruitment.

A third and final explanation for the absence of age differences in the present results is related to the behavioral results. Increased cortical recruitment, possibly reflecting a compensatory mechanism, has been reported in older adults with high or age-unaffected cognitive performance (Cabeza et al., 2002; Eyler et al., 2011). While older adults in this study generally demonstrated lower performance in all conditions, including the perceptual baseline control task, this age-related effect did not interact with condition. Older adults were not disproportionately affected in the selective attention conditions as compared to baseline. These age differences are therefore more likely to reflect generalized mental slowing, which is well documented in the aging literature (Salthouse, 1985; Krail and Salthouse, 1994). Consequently, our finding that neural modulation during cross-modal selective attention does not differ between older and younger individuals most likely does not merely reflect an inability of older participants to employ compensatory mechanisms.

An important limitation to our study may be the sample size. However, inspection of probabilistic maps of all comparisons, showing similar activation patterns and no regions that stand out in any age group, may point toward true age-independent attentional modulation, and not merely insufficient power for detecting age-related differences. Moreover, age group effects of attentional modulation have been detectable in previous studies with as little as ten participants per age group (Townsend et al., 2006). Nevertheless, it is warranted that our findings of age-equivalent top-down modulation during cross-modal selective attention be further substantiated with larger samples.

In conclusion, the present study provided more insight into the neural basis of cross-modal visual and auditory selective attention by demonstrating that key brain regions that have been extensively documented to be involved in, mainly visual, unimodal selective attention are also recruited for cross-modal visual and auditory selective attention. In addition, we have shown patterns of activity that suggest dominance of visual over auditory processing at the neural level. Most importantly, however, we have demonstrated that top-down modulation in the setting of cross-modal selective attention is independent of age, which suggests intact neural functioning of this crucial cognitive function in older age.

## Data Availability Statement

The datasets analyzed in this manuscript are not publicly available. Requests to access the datasets should be directed to franziska.rienacker@maastrichtuniversity.nl.

## Ethics Statement

The studies involving human participants were reviewed and approved by Ethics Review Committee Psychology and Neurosciences (ERCPN), Maastricht University. The patients/participants provided their written informed consent to participate in this study.

## Author Contributions

MG and PV designed the experimental paradigm. MG gathered the fMRI and behavioral data. FR executed the analyses and wrote the manuscript. MG, PV, HJ, and CV contributed to the conception of the study, interpretation of the results, and preparation of the manuscript. MG, JE, and HJ supported the fMRI analyses. All authors contributed to the article and approved the submitted version.

## Conflict of Interest

The authors declare that the research was conducted in the absence of any commercial or financial relationships that could be construed as a potential conflict of interest.
