# Supplementary material for: The Neural Correlates of Visual and Auditory Cross-Modal Selective Attention in Aging
Source: Front Aging Neurosci. 2020 Nov 12;12:498978. doi: 10.3389/fnagi.2020.498978 (PMC7693624; doi:10.3389/fnagi.2020.498978)
Supplement: Supplementary file 1 [file Table_1.DOCX]

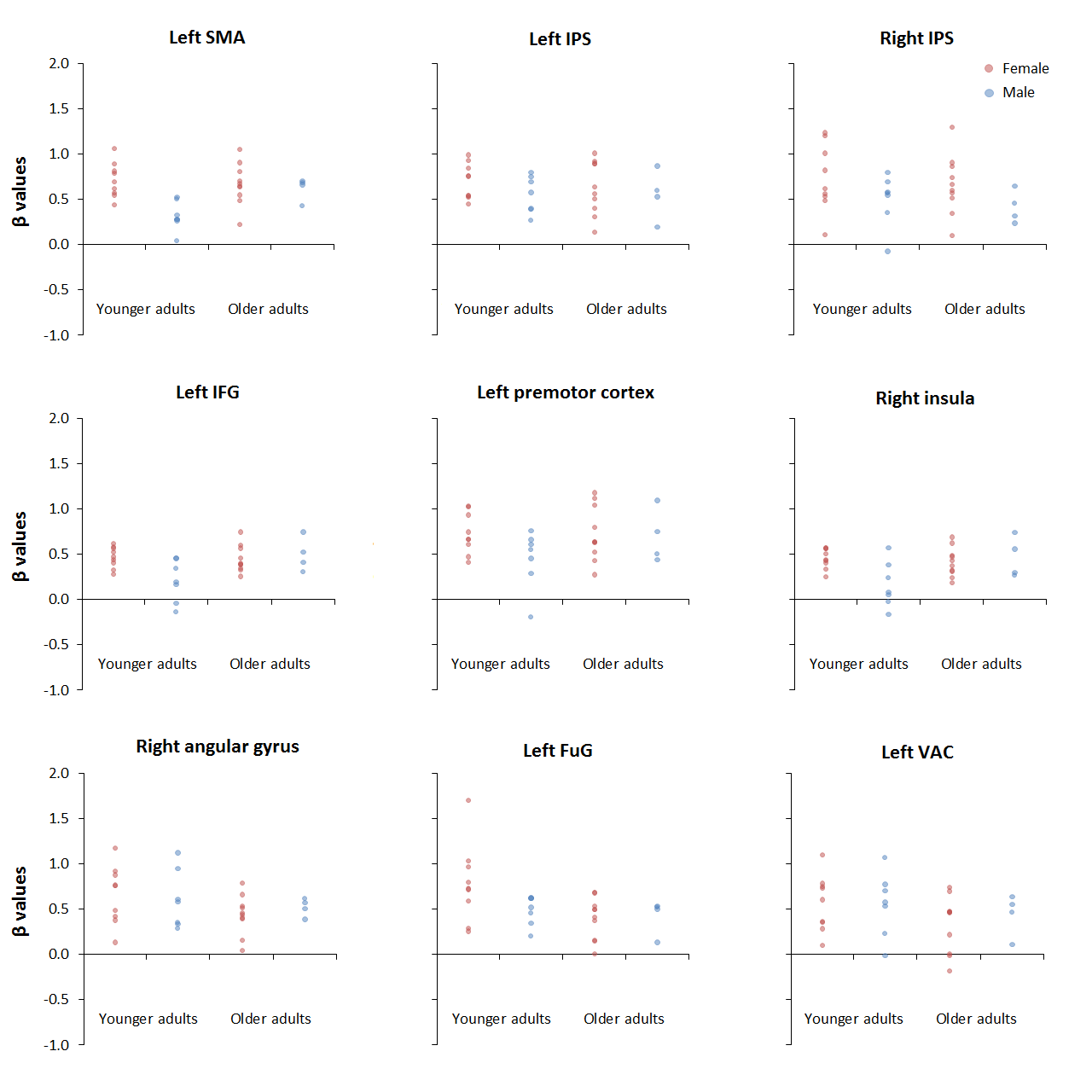


**Supplementary Figure 1.** *β* values for each participant, as a function of age group and sex, in each of the regions showing significant attentional modulation during cross-modal visual attention. SMA = supplementary motor area; IPS = intraparietal sulcus; IFG = inferior frontal gyrus; FuG = fusiform gyrus; VAC = visual association cortex.


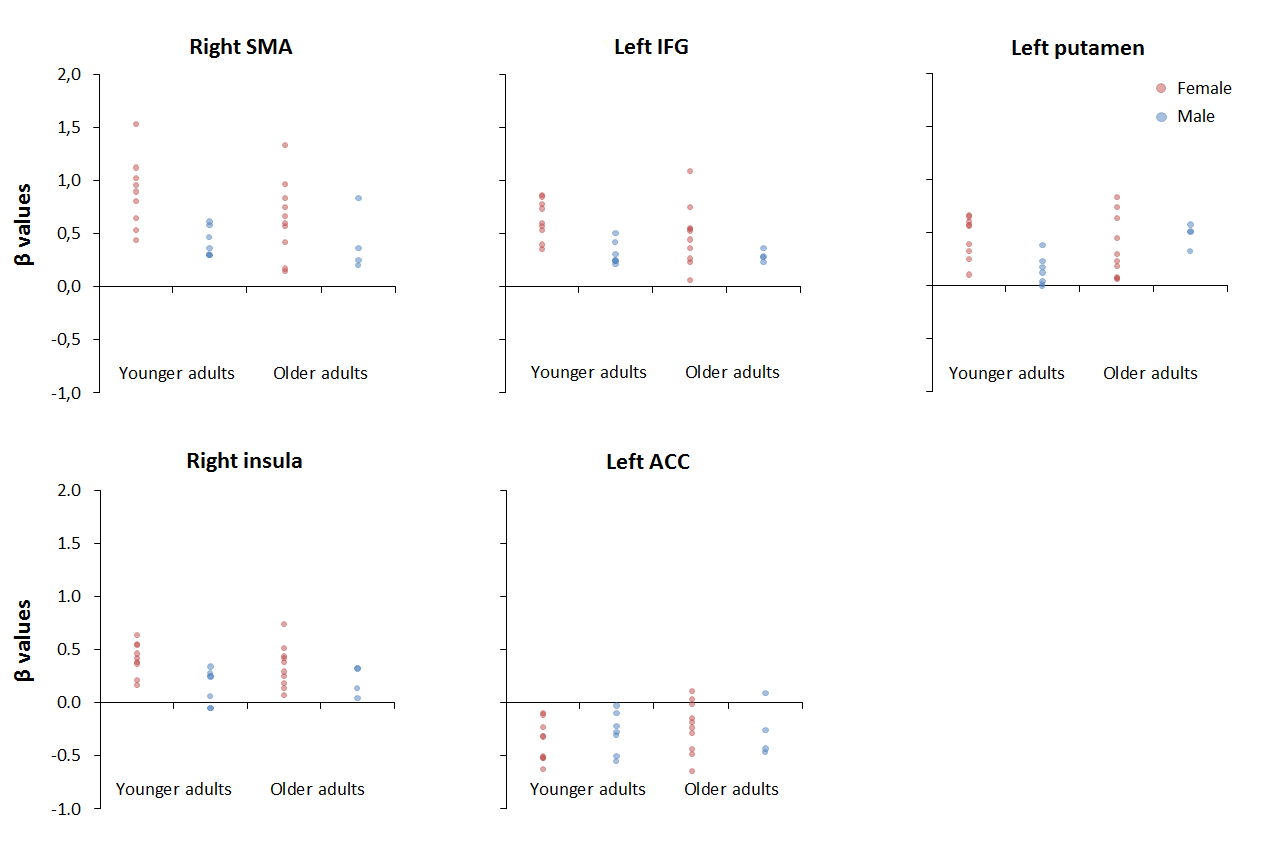


**Supplementary Figure 2.** *β* values for each participant, as a function of age group and sex, in each of the regions showing significant attentional modulation during cross-modal auditory attention. SMA = supplementary motor area; = inferior frontal gyrus; ACC = anterior cingulate cortex.

**
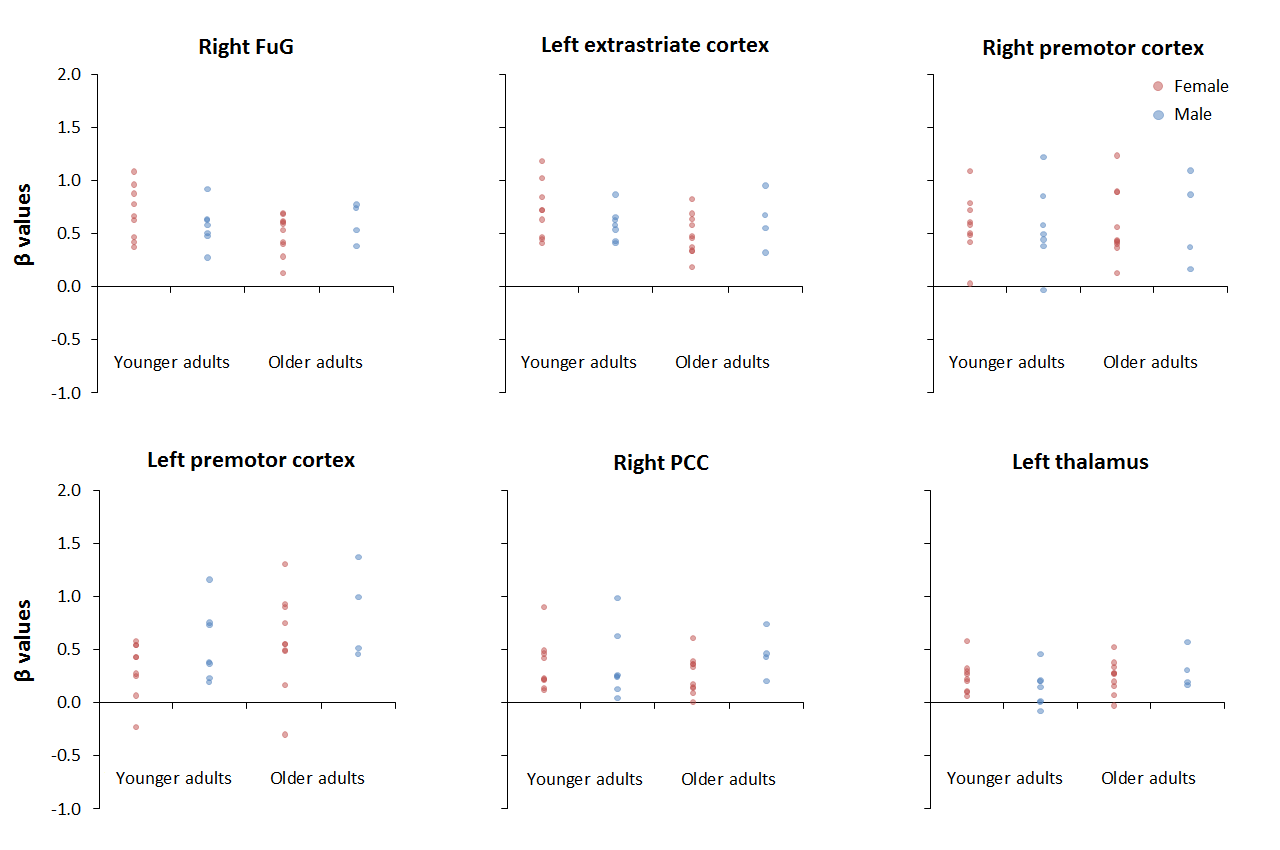
**

**Supplementary Figure 3.** *β* values for each participant, as a function of age group and sex, in each of the regions showing significant differences in attentional modulation across sensory modalities. FuG = fusiform gyrus; PCC = posterior cingulate cortex.
